# A case series of advanced renal cell carcinoma patients treated with neoadjuvant cabozantinib prior to cytoreductive nephrectomy within the phase 2 CABOPRE trial

**DOI:** 10.18632/oncotarget.27807

**Published:** 2020-11-24

**Authors:** Guillermo de Velasco, Lucia Carril-Ajuria, Felix Guerrero-Ramos, Teresa Alonso-Gordoa, Juan F. Rodríguez-Moreno, Alberto Carretero, Maricruz Martin-Soberon, Federico de la Rosa-Kehrmann, Daniel Castellano

**Affiliations:** ^1^Medical Oncology Department, Hospital Universitario 12 de Octubre, Comunidad de Madrid, Madrid, Spain; ^2^Urology Department, Hospital Universitario 12 de Octubre, Comunidad de Madrid, Madrid, Spain; ^3^Medical Oncology Department, Hospital Universitario Ramón y Cajal, Comunidad de Madrid, Madrid, Spain; ^4^Medical Oncology Department, Hospital Universitario HM Sanchinarro, Comunidad de Madrid, Madrid, Spain

**Keywords:** kidney cancer, renal cell carcinoma, neoadjuvant treatment, cabozantinib, trial

## Abstract

Cytoreductive nephrectomy has long been used to improve disease control in metastastic Renal Cell Carcinoma (mRCC). However, based on the results of the CARMENA and SURTIME trials, cytoreductive nephrectomy is no longer the standard of care in patients requiring upfront systemic treatment and it should be avoided in most poor-risk patients. Nevertheless, it should still be considered in patients responding to systemic therapy and good-risk patients not requiring systemic treatment.

This case series of the phase 2 CABOPRE trial suggests neoadjuvant cabozantinib may be able to induce rapid and significant responses in some intermediate-risk advanced renal cell carcinoma patients facilitating resectability.

## INTRODUCTION

Systemic treatment (ST) has been the cornerstone of advanced renal cell carcinoma (aRCC) but CN has also long been used to improve disease control [1–5]. Currently, the role of CN for aRCC patients has yet to be defined. The SURTIME and the CARMENA trials provided new data but the definitive role of CN remains unclear [6, 7]. The SURTIME trial, suggested the non-inferiority of deferred CN compared to upfront CN [7]. The CARMENA trial demonstrated the non-inferiority of sunitinib alone compared to immediate CN followed by sunitinib [6]. These results support that CN should no longer be the standard of care in patients requiring ST upfront and suggest that surgery should be avoided in IMDC International Metastatic RCC database Consortium-(IMDC)-poor risk patients. Nevertheless, CN could remain as an option for selected patients responding to ST and good-risk patients not requiring ST [8].

Within this context the CABOPRE trial (EudraCT Number: 2018-001201-93) was designed as an open-arm phase II trial, to assess the efficacy and safety of cabozantinib 60 mg/daily, given as perioperative treatment for aRCC patients in potential candidates to CN. The primary endpoint was objective response rate (ORR) by RECIST 1.1 (Response Criteria Evaluation Criteria in Solid Tumors) at 12 weeks. The goal of this case series is to illustrate the preliminary experience of the CABOPRE trial with some illustrative cases of aRCC treated with neoadjuvant (NA) cabozantinib prior to CN.

### Patient 1: Tumour thrombus downsize in a locally advanced (LA) RCC patient treated with NA cabozantinib

A 60 year-old man with no relevant medical history, presented in the Emergency Department (ED) with back pain and hematuria in March of 2019. A thoracoabdominal-pelvic (TAP) computed tomography (CT) scan revealed a left renal mass of 9 cm with renal vein and inferior cave vein (ICV) invasion, paraaortic lymph nodes and small bilateral lung metastases. A CT guided biopsy of the renal mass revealed a Fuhrman grade 3 clear cell RCC (ccRCC).

**Figure 1:**
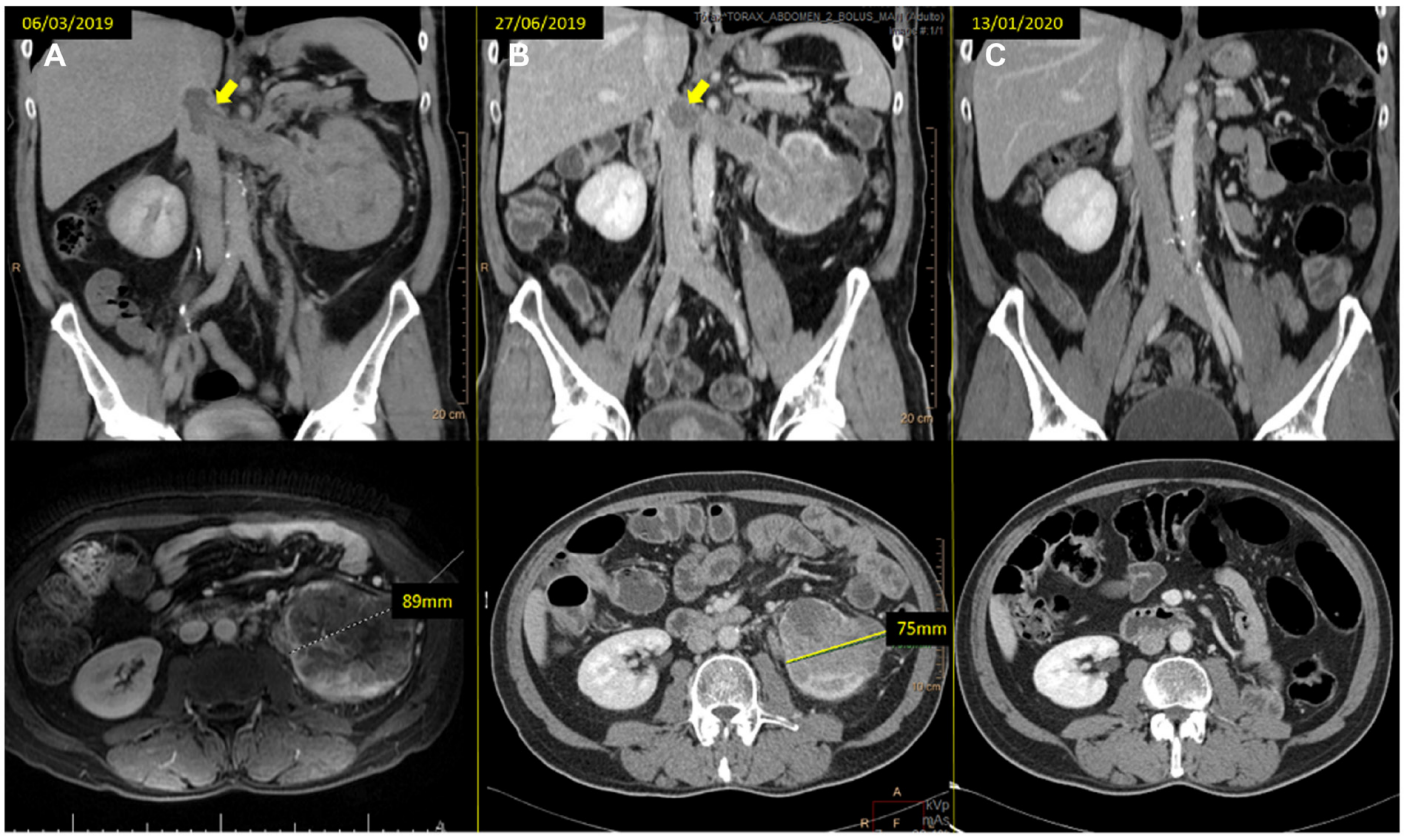
(**A**) baseline CT scan and (**B**) the reassessment CT scan at 12 weeks of treatment, showing the tumor thrombus downsizing (arrows) and the left kidney mass response (from 89 to 75 mm). (**C**) last reassessment CT scan where the patient remains progression free.

The patient was classified as intermediate-risk by IMDC and was enrolled in the CABOPRE trial. The patient underwent three cycles of full dose cabozantinib (60 mg/d) with acceptable tolerance and achieving a significant downsizing of the tumor thrombus and renal mass (Figure 1). Radical nephrectomy and IVC/renal vein thrombectomy were successfully performed, confirming a pT3bNxMx ccRCC with IVC/left renal vein extension. The patient restarted cabozantinib at a dose of 40 mg daily after surgery and has remained progression free to date (follow-up 12 months). The benefits of tumor thrombus reduction are well known, reducing surgical morbidity and mortality and enabling a complete surgical resection of both the primary tumor and the tumor thrombus which is key to improve survival outcomes [9].

### Patient 2: Rapidly progressive aRCC during cabozantinib break despite initial response

**Figure 2:**
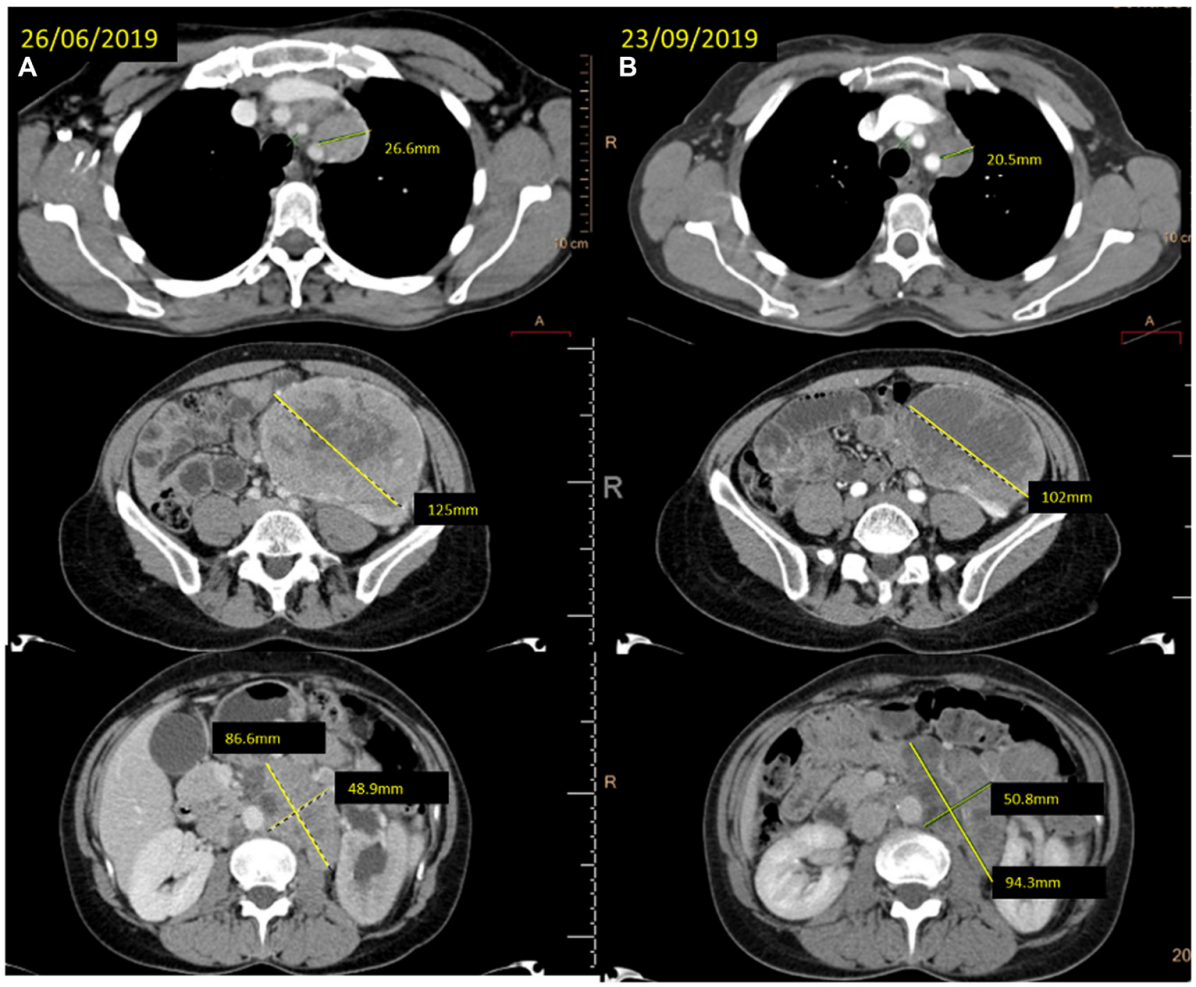
(**A**) baseline CT scan and (**B**) the reassessment CT scan showing the response achieved after 12 weeks of treatment with cabozantinib at different levels (mediastinal lymph nodes, left kidney mass and retroperitoneal lymph nodes).

A 46 year-old woman current smoker and with no other relevant medical history presented in the ED in May 2019 with a palpable abdominal mass and intense right clavicle pain. A TAP CT scan was performed showing a large left renal mass of ~17.5 cm in its long axis and revealing mediastinal/ retroperitoneal lymph node and bone metastasis (right clavicle), as well as left lung nodules suspicious of metastases. CT guided biopsy confirmed Fuhrman grade 3 ccRCC. The patient was classified as intermediate-risk by IMDC and met all criteria of the CABOPRE trial. She underwent three cycles before surgery and required dose reduction to 40 mg daily from the third cycle due to grade 2 mucositis. No other toxicity was reported, and the reassessment CT scan demonstrated a significant response of both the renal mass and the mediastinal and retroperitoneal lymph nodes metastatic disease (Figure 2). Radical nephrectomy with left adrenalectomy and lymphadenectomy was performed in October 2019 without acute complications. Unfortunately, the CT scan performed 12 weeks later revealed lymph node, lung and hepatic progression.

Timing of CN is not defined. Median time to response to cabozantinib is around 1.9 months [10]. Thus, our trial was designed for 12 weeks of neoadjuvant cabozantinib before CN. Regrettably, not all patients who respond to systemic therapy may benefit from CN. In fact, our patient presented progressive disease after 3 cycles of the ipilimumab/nivolumab combination. A third line therapy with a TKI again (Axitinib) was attempted without benefit. Cancer treatment was finally stopped, and the patient is currently under best supportive care.

### Patient 3: Resectable kidney mass after response to NA cabozantinib, but unfortunately medically inoperable

**Figure 3:**
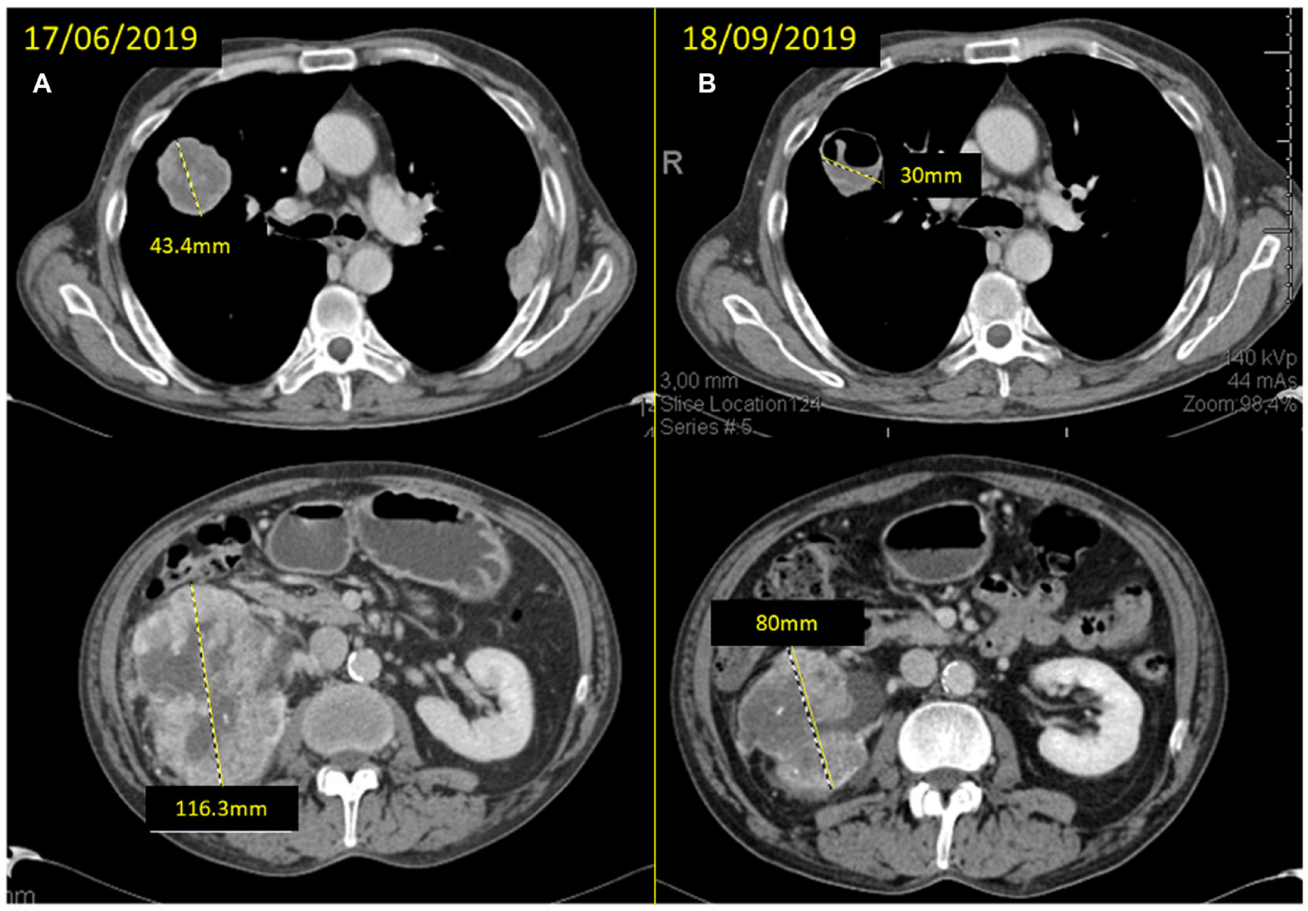
(**A**) baseline CT scan and (**B**) response after 12 weeks of neoadjuvant treatment with cabozantinib with significant reduction of the mass in the superior right lobe (SRL) from 43.4 to 30 mm and of the right kidney mass from 116.3 to 80 mm.

A 59 year-old man current smoker and with chronic obstructive pulmonary disease (COPD), presented in the ED with sharp left costal pain. A thoracic radiography demonstrated cannonball metastases with a pleural thickening of 6 cm on the upper left lobe, a right renal mass of 16 cm and right iliac metastasis with soft tissue extension. Bronchoscopy was performed and the biopsies revealed a Fuhrman grade 2 ccRCC. The patient was classified as intermediate-risk by IMDC and was included in our study. The patient underwent three cycles of cabozantinib with good tolerance and achieving partial response by RECIST 1.1 (Figure 3). After multidisciplinary team discussion, although resectable, the patient was finally considered inoperable due to worsening of his COPD. Cabozantinib was continued at 60 mg/day. Unfortunately, two months later the patient presented clinical progression with tumoral hypercalcemia and radiological progression with kidney mass enlargement and new bone lesions on the reassessment CT scan. In this case the response was deeper in the metastases than in the primary as predictable. The key point that illustrates this case is whether a CN would have impacted the prognosis of this patient.

### Patient 4: Maintained disease control with cabozantinib allows successful CN and lung metastasectomy approach

**Figure 4:**
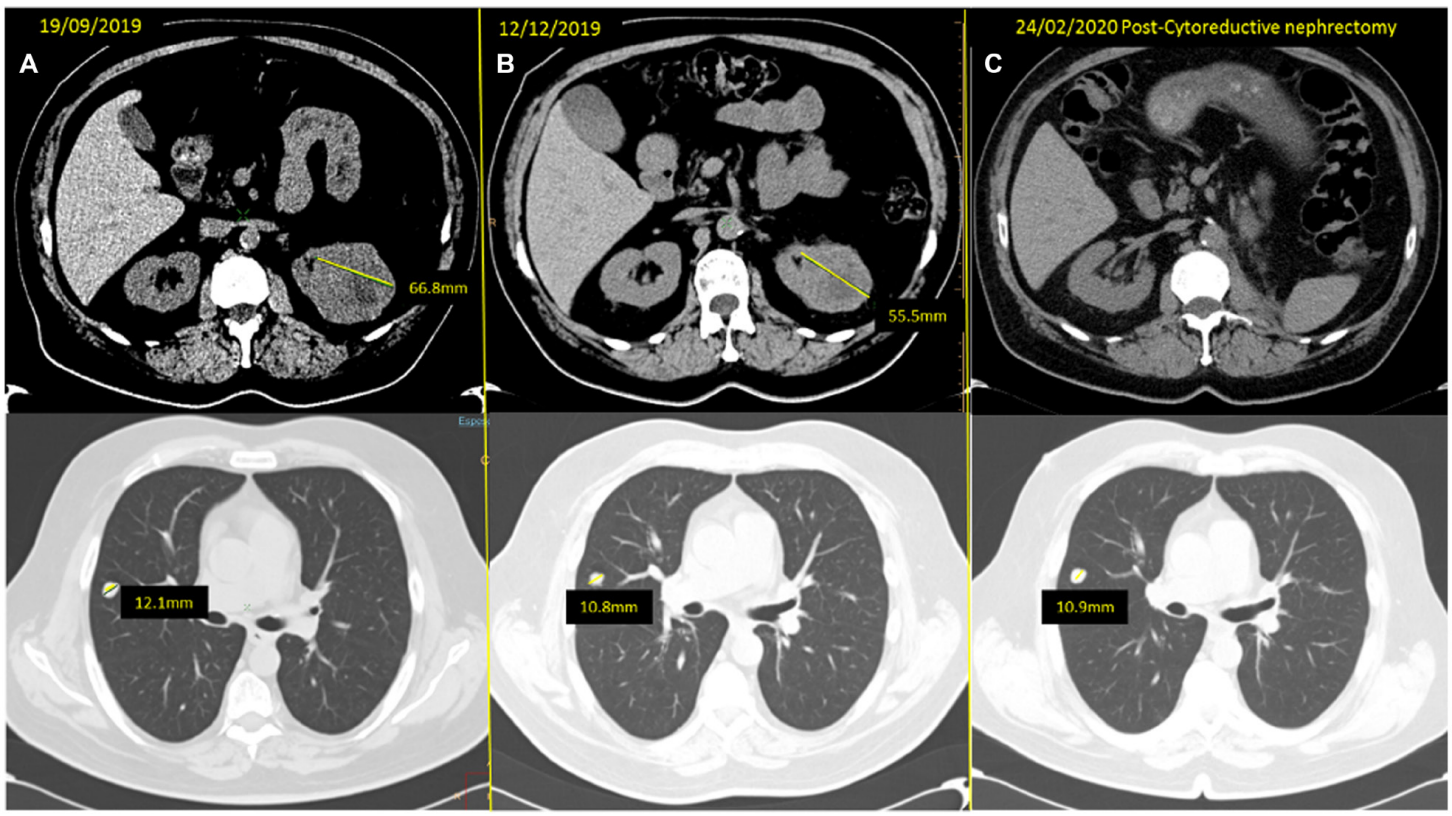
(**A**) baseline CT scan and (**B**) response obtained after three months of neoadjuvant cabozantinib, with a reduction of the left kidney mass from 66.8 to 55.5 mm and of the SRL nodule from 12.1 to 10.8 mm. (**C**) Last reassessment CT scan, after CN, showing only stability of the remaining solitary lung metastases which is pending surgery.

A 60 year-old man with a medical history of hypertension, type 2 diabetes mellitus and coronary artery disease, was diagnosed of a left kidney renal mass by ultrasound due to intermittent hematuria. A TAP CT scan was then performed demonstrating a left kidney mass of 66.8 mm and small bilateral lung nodules, being the largest of 12 mm. CT guided biopsy of both the left kidney mass and the lung nodule revealed a Fuhrman grade 1 ccRCC. The patient received 12 weeks of cabozantinib requiring dose reduction to 40 mg daily after the first cycle due to grade 2 asthenia. The reassessment CT scan showed a reduction of both the primary tumor and the lung metastasis but without achieving PR by RECIST 1.1 criteria (–16%) (Figure 4). Left kidney CN was performed successfully on December 2019. Cabozantinib at dose of 40 mg daily was reinitiated three weeks later. Two months later the treatment was discontinued due to poor tolerability (dysphonia, diarrhea and anxiety) and maximal benefit achieved with stability of the target lung nodule metastasis. The patient is currently waiting for surgical resection of the only solitary lung visible metastasis which has been delayed due to the COVID19 pandemic.

## DISCUSSION

Cabozantinib is a multi-kinase inhibitor (vascular endothelial growth factor receptor 2, tyrosine-protein kinases (TKI) MET and AXL) that has shown better disease control rate (DCR) and response rate (RR) than first generation TKIs such as sunitinib (i.e., DCR of 75% vs 47% and RR of 20% vs 9% respectively, CABOSUN trial) [10]. These results anticipated an increased RR when given prior to nephrectomy, which added to the short time-to-response reported with this drug, make it suitable for perioperative treatment. Here, we have reported the outcomes of four patients treated in the CABOPRE study as preliminary data of the trial. Remarkably, although all these patients were intermediate-risk and most of them had high tumor burden and high number of comorbidities, the tumor shrinkage was remarkable. Two (Patients 2–3) of the four cases achieved partial response by RECIST 1.1. criteria, and the other two patients presented a significant response. Patient 1 presented a significant downsizing of the tumor thrombus which facilitated surgical resectability and decreased risk of postoperative complications, and patient 4 achieved a sustained stability enabling a successful CN which will be followed by lung metastasectomy. Surprisingly, there is still no single predictive biomarker developed for RCC. This perioperative treatment with active drugs allows better selection of patients for CN and precludes a perfect window of opportunity for translational research in order to optimize treatment in the near future (multiple translation studies are ongoing).

On the downside, these preliminary results raise concerns about the breaks, either surgery-related or due to toxicity, and disease progression, as well as the perfect timing for delayed CN. Recently, the combination of immune checkpoint inhibitors (IO) with either TKI or double IO-IO has shown an improvement in RR and overall survival [4, 5]. CN may still have a role, especially with these novel combos since the response of the primary tumor is usually less intense than of the metastases [11].

In conclusion, cabozantinib as perioperative treatment can induce rapid and remarkable responses in intermediate-risk patients facilitating resectability in advanced or metastatic ccRCC patients. These preliminary results are promising, and definitive results and biomarkers of this trial are awaited. Final results will be reported as a global efficacy data including the CN rate and the relationship with tumor-shinkrage.

## References

[1] Escudier B , Porta C , Schmidinger M , Rioux-Leclercq N , Bex A , Khoo V , Grünwald V , Gillessen S , Horwich A ; ESMO Guidelines Committee. Renal cell carcinoma: ESMO Clinical Practice Guidelines for diagnosis, treatment and follow-up^†^. Ann Oncol. 2019; 30:706–720. 10.1093/annonc/mdz056. 30788497

[2] Flanigan RC , Salmon SE , Blumenstein BA , Bearman SI , Roy V , McGrath PC , Caton JR Jr , Munshi N , Crawford ED . Nephrectomy followed by interferon alfa-2b compared with interferon alfa-2b alone for metastatic renal-cell cancer. N Engl J Med. 2001; 345:1655–1659. 10.1056/NEJMoa003013. 11759643

[3] Albiges L , Powles T , Staehler M , Bensalah K , Giles RH , Hora M , Kuczyk MA , Lam TB , Ljungberg B , Marconi L , Merseburger AS , Volpe A , Abu-Ghanem Y , et al. Updated European Association of Urology Guidelines on Renal Cell Carcinoma: Immune Checkpoint Inhibition Is the New Backbone in First-line Treatment of Metastatic Clear-cell Renal Cell Carcinoma. Eur Urol. 2019; 76:151–156. 10.1016/j.eururo.2019.05.022. 31151678

[4] Motzer RJ , Tannir NM , McDermott DF , Arén Frontera O , Melichar B , Choueiri TK , Plimack ER , Barthélémy P , Porta C , George S , Powles T , Donskov F , Neiman V , et al; CheckMate 214 Investigators. Nivolumab plus Ipilimumab versus Sunitinib in Advanced Renal-Cell Carcinoma. N Engl J Med. 2018; 378:1277–1290. 10.1056/NEJMoa1712126. 29562145 PMC5972549

[5] Rini BI , Plimack ER , Stus V , Gafanov R , Hawkins R , Nosov D , Pouliot F , Alekseev B , Soulières D , Melichar B , Vynnychenko I , Kryzhanivska A , Bondarenko I , et al; KEYNOTE-426 Investigators. Pembrolizumab plus Axitinib versus Sunitinib for Advanced Renal-Cell Carcinoma. N Engl J Med. 2019; 380:1116–1127. 10.1056/NEJMoa1816714. 30779529

[6] Méjean A , Ravaud A , Thezenas S , Colas S , Beauval JB , Bensalah K , Geoffrois L , Thiery-Vuillemin A , Cormier L , Lang H , Guy L , Gravis G , Rolland F , et al. Sunitinib Alone or after Nephrectomy in Metastatic Renal-Cell Carcinoma. N Engl J Med. 2018; 379:417–427. 10.1056/NEJMoa1803675. 29860937

[7] Bex A , Mulders P , Jewett M , Wagstaff J , van Thienen JV , Blank CU , van Velthoven R , del Pilar Laguna M , Wood L , van Melick HHE , Aarts MJ , Lattouf JB , Powles T , et al. Comparison of Immediate vs Deferred Cytoreductive Nephrectomy in Patients With Synchronous Metastatic Renal Cell Carcinoma Receiving Sunitinib: The SURTIME Randomized Clinical Trial. JAMA Oncol. 2019; 5:164–170. 10.1001/jamaoncol.2018.5543. 30543350 PMC6439568

[8] Bhindi B , Graham J , Wells JC , Bakouny Z , Donskov F , Fraccon A , Pasini F , Lee JL , Basappa NS , Hansen A , Kollmannsberger CK , Kanesvaran R , Yuasa T , et al. Deferred Cytoreductive Nephrectomy in Patients with Newly Diagnosed Metastatic Renal Cell Carcinoma. Eur Urol. 2020; 78:615–623. 10.1016/j.eururo.2020.04.038. 32362493

[9] Quencer KB , Friedman T , Sheth R , Oklu R . Tumor thrombus: incidence, imaging, prognosis and treatment. Cardiovasc Diagn Ther. 2017; 7:S165–S177. 10.21037/cdt.2017.09.16. 29399520 PMC5778532

[10] Choueiri TK , Hessel C , Halabi S , Sanford B , Michaelson MD , Hahn O , Walsh M , Olencki T , Picus J , Small EJ , Dakhil S , Feldman DR , Mangeshkar M , et al. Cabozantinib versus sunitinib as initial therapy for metastatic renal cell carcinoma of intermediate or poor risk (Alliance A031203 CABOSUN randomised trial): Progression-free survival by independent review and overall survival update. Eur J Cancer. 2018; 94:115–125. 10.1016/j.ejca.2018.02.012. 29550566 PMC6057479

[11] Albiges L , Tannir N , Burotto M , McDermott DF , Plimack ER , Barthélémy P , Porta CG , Powles TB , Donskov F , George S , Kollmannsberger C , Gurney H , Grimm MO , et al 711P Nivolumab + ipilimumab (N+I) vs sunitinib (S) for first-line treatment of advanced renal cell carcinoma (aRCC) in CheckMate 214: 4-year follow-up and subgroup analysis of patients (pts) without nephrectomy. Ann Oncol. 2020; 31:S559–60. 10.1016/j.annonc.2020.08.783.

