# Addendum: A case series of advanced renal cell carcinoma patients treated with neoadjuvant cabozantinib prior to cytoreductive nephrectomy within the phase 2 CABOPRE trial

**DOI:** 10.18632/oncotarget.28263

**Published:** 2022-08-04

**Authors:** Guillermo de Velasco, Lucia Carril-Ajuria, Felix Guerrero-Ramos, Teresa Alonso-Gordoa, Juan F. Rodríguez-Moreno, Alberto Carretero, Maricruz Martin-Soberon, Federico de la Rosa-Kehrmann, Daniel Castellano

**Affiliations:** ^1^Medical Oncology Department, Hospital Universitario 12 de Octubre, Comunidad de Madrid, Madrid, Spain; ^2^Urology Department, Hospital Universitario 12 de Octubre, Comunidad de Madrid, Madrid, Spain; ^3^Medical Oncology Department, Hospital Universitario Ramón y Cajal, Comunidad de Madrid, Madrid, Spain; ^4^Medical Oncology Department, Hospital Universitario HM Sanchinarro, Comunidad de Madrid, Madrid, Spain

**Consent was added to this article:** All patients signed informed consent.

Original article: Oncotarget. 2020; 11:4457–4462. 4457-4462. https://doi.org/10.18632/oncotarget.27807

